# Comfort and Usability of Digital Versus Conventional Custom‐Fit Mouthguards: A Randomized Clinical Trial

**DOI:** 10.1111/edt.70059

**Published:** 2026-01-28

**Authors:** Maryuri Macías Bautista, Priscilla Barbosa Ferreira Soares, Airin Karelys Avendaño Rondon, Laryssa Silva da Cunha, Ianca Daniele Oliveira de Jesus, Rodrigo Silva Moreira, Gisele Rodrigues da Silva, Liran Levin, Carlos José Soares

**Affiliations:** ^1^ Department of Dentistry and Dental Materials, Faculty of Dentistry Federal University of Uberlândia Uberlândia Minas Gerais Brazil; ^2^ Department of Periodontics and Implantology, Faculty of Dentistry Federal University of Uberlândia Uberlândia Minas Gerais Brazil; ^3^ College of Dentistry University of Saskatchewan Saskatoon Saskatchewan Canada

**Keywords:** 3D printing, athlete injuries, dental trauma, protective equipment, sports dentistry

## Abstract

**Background/Objectives:**

The use of mouthguards protects teeth and the supporting tissues against impacts. Digital impressions and 3D‐printed models can reduce manufacturing steps and minimize discomfort. This study evaluated the fit, comfort, and usability of custom‐made mouthguards, obtained by conventional and digital workflow, in amateur athletes.

**Materials and Methods:**

Amateur athletes were recruited for this randomized, double‐blind, crossover clinical study. Each participant received two 4‐mm‐thick custom‐fit mouthguards made of ethylene vinyl acetate (EVA) sheets using two protocols: conventional impression using alginate to produce dental stone cast (CMP) and intraoral digital scanning to produce 3D‐printed resin models (DMP). Each mouthguard was worn during all training and matches over 3 months. The order of mouthguard use was randomly assigned. Patient satisfaction with the impression protocol and mouthguard use, model accuracy, and mouthguard fit were assessed.

**Results:**

Overall, 46 participants completed the study. The DMP protocol required less execution time (*p* = 0.023), caused less discomfort (*p* = 0.018), anxiety (*p* = 0.008), nausea (*p* = 0.027), difficulty breathing (*p* < 0.001), and unpleasant taste (*p* = 0.006) compared to the CMP protocol. The pain (*p* = 0.971) perceived by the participants was similar in both impression protocols. The CMP‐mouthguards were considered to be better fitting by the majority of participants (*p* = 0.027). Between‐group comparisons revealed that DMP‐mouthguard caused less discomfort immediately postadaptation (*p* = 0.034), with no significant differences observed at 30 or 90 days.

**Conclusions:**

The digital workflow demonstrated to be efficient in the fabrication of mouthguards. Digital impression was more comfortable, requiring less execution time, with more accurate printed models and produced mouthguards with reported comfort and fit levels similar to those manufactured conventionally.

## 1 Introduction

Involvement in sports is universal, but the nature of some sports creates the potential for dental trauma [1, 2]. Younger and amateur athletes are at greater risk of oral injuries, as they may not receive proper guidance and training [3]. Sports injuries represent one‐third of oral and craniofacial injuries [4] and can be considered a public oral health problem due to the impact on the daily life of the athlete and caregivers, in addition to the cost of treatments [5, 6].

The use of mouthguards in sports has been shown to reduce the risk of orofacial injuries [7, 8, 9, 10, 11, 12, 13, 14, 15, 16], as they absorb the energy generated by the impact and reduce stress and tension transmitted to the teeth and supporting tissues [17, 18]. The use of mouthguards also protects against mandibular condyles fractures [19], and reduces traumatic impact on the temporomandibular joint [20, 21, 22]. The World Dental Federation (FDI) and the International Association of Dental Traumatology (IADT) recommend the use of mouthguards in the practice of any contact sport [3, 23]. Custom‐fit mouthguards offer protection against intraoral injuries due to the resilience they provide, being capable of distributing and dissipating stress and tension resulting from impact forces [2, 17]. When properly manufactured and used, custom‐fit mouthguards promote better adaptation, retention and comfort [24, 25], and in some reports optimize sports performance [11, 14, 26]. They offer better fit, stability, breathing capacity, phonetics, and protection of dental structures [13, 18], and adjacent tissues compared to the prefabricated mouthguards [20, 24].

The mechanical performance of mouthguard is affected by several factors, such as the type of material, geometry, manufacturing process, thickness, and time of use [12, 14]. These factors also influence fit, comfort, and usability [26, 27]. Ethylene‐vinyl acetate (EVA) is the most commonly used material for mouthguards manufacturing [28], due to its ease of molding and handling [29]. Through thermoplasticization, it can be used to create custom‐fit mouthguards using working templates [8, 30].

The mouthguard is manufactured using the conventional process, which begins with the impression of the athlete's dental arches, usually made with alginate or polyvinyl siloxane [7, 13]. The mold is then poured, and a dental stone cast working model is usually obtained [12, 27]. However, this process causes discomfort during impression taking and, due to the multiple steps involved, may lead to structural deformation of both the impression and the model, interfering with the adaptation of the mouthguard [31, 32, 33].

The use of a digital workflow for manufacturing mouthguard is an innovative method, as it reduces the number of steps and allows for greater reproducibility [12, 13, 15, 31]. Intraoral scanning of the arches and occlusal registrations are converted into digital stereolithography (STL) files that are used to manufacture 3D‐printed resin models, on which the mouthguard is produced by plasticizing EVA [12, 33, 34]. This method offers the advantage of reducing discomfort for patients who are reluctant to undergo traditional impression techniques [32, 35].

Although there are laboratory studies that comparatively evaluate the fabrication of mouthguards obtained by conventional and digital methods [12, 13], there is a lack of clinical studies in the literature that evaluate the impact of digital scanning and the fabrication of 3D‐printed resin models on the fit, comfort, and usability of custom‐fit mouthguards made with EVA. Therefore, the objective of this study was to evaluate these parameters on custom‐fit EVA mouthguards obtained by conventional and digital workflows in amateur athletes. The null hypothesis was that digital scanning and the use of printed models for the fabrication of custom‐fit mouthguards would not influence the fit, comfort, or usability of mouthguards in the population evaluated.

## 2 Materials and Methods

**FIGURE 1:**
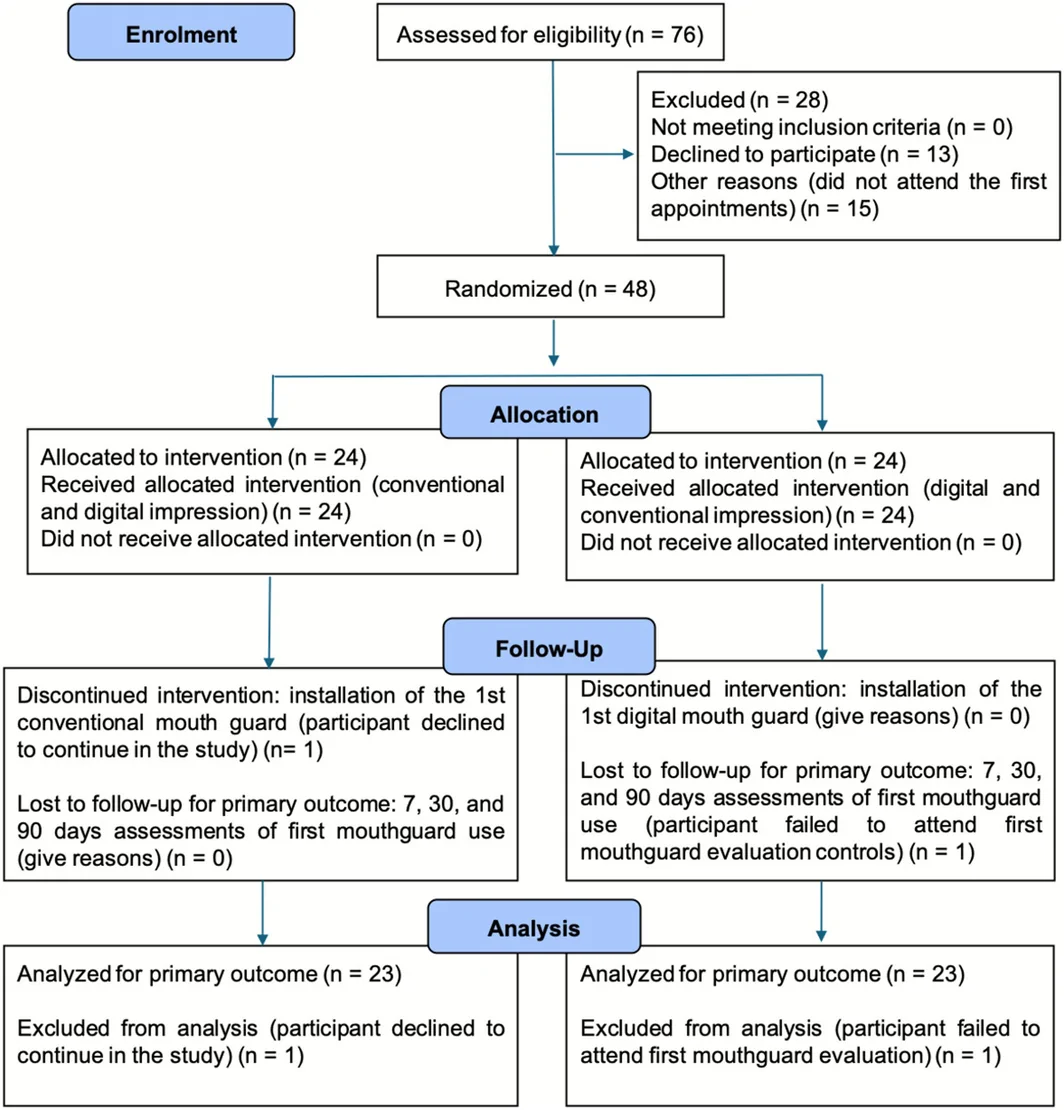
CONSORT 2025 flow diagram: Flowchart diagram of the progress through the phases of a randomized trial of two groups (i.e., enrolment, intervention allocation, follow‐up, and data analysis) [36].

This randomized, crossover, double‐blind clinical trial was approved by the Ethics Committee of the Federal University of Uberlândia (CAAE: 77752824.4.0000.5152) and registered in the Brazilian Clinical Trials Registry (UTN: U1111‐1316‐7546; URL: REBEC). The study followed the CONSORT protocol [36] (Supporting Information) and was conducted according to the flowchart presented in Figure 1. The primary outcomes were comfort and usability to use mouthguard expressed by anxiety, nausea, difficulty breathing, and unpleasant taste. The secondary outcomes were the time to produce mouthguard, pain during the use, and fit of the mouthguards.

The sample size was determined using G*Power software (Version 3.1.9), following as reference a similar crossover study [37]. The *α* was established at 0.05 and the *β* 0.80. The effect size assumed was 0.4 and indicated a sample size of 41 participants. To account for potential withdrawals and refusals, a total of 48 amateurs' athletes were recruited. To recruit participants, an invitation was made at two multisports centers in the city of Uberlandia to athletes who predominantly practice contact sports or are at risk of dentoalveolar trauma and who could use a mouthguard.

The inclusion criteria were: athletes aged 16 years or older, with sufficient critical reasoning to answer the questionnaire objectively, that they practiced some sport regularly for at least twice per week and that they agreed to use a mouthguard in all sports activities. Athletes who had suffered recent dentoalveolar trauma, presented pain, root fracture, alveolar or condylar, requiring dental containment, or some chronic illness with oral manifestations were excluded.

In the first session, a questionnaire was applied to obtain information on gender, age, sport practice, training time, usual position in the game, previous experience with orofacial injury and experience with the use of a mouthguard, in addition to knowledge about the importance of using a mouthguard. To evaluate the questionnaires applied in this investigation, they were previously tested in a population similar to that of the sample, with participants who did not participate in the study [38]. All participants underwent two impression protocols in the same session, involving both conventional and digital impressions for the fabrication of two custom‐made mouthguards. Each impression protocol (conventional and digital) was timed, including any repetitions required during the procedures, and the tray individualization process was also included for the conventional protocol. The order in which the protocols were performed for each participant was determined through randomization (https://www.random.org/lists/).

To assign which participants would be treated by each operator throughout all procedures, the participants and operators were divided into two equally sized, randomized groups. From start to finish, the same operator performed both the conventional and digital impression protocols as well as the fitting and adjustment of both mouthguards at the designated time points. This approach aimed to minimize any potential interference with the subsequent perceptual evaluations made by the participants. The operators were two doctoral students with previous experience in conventional impression taking and intraoral scanning for the manufacture of mouthguards. Both operators were previously calibrated in performing each impression protocol, conventional and digital, as well as in the subsequent adaptation and adjustment of both types of mouthguards, using a pilot sample group that did not participate in the main study.

**FIGURE 2:**
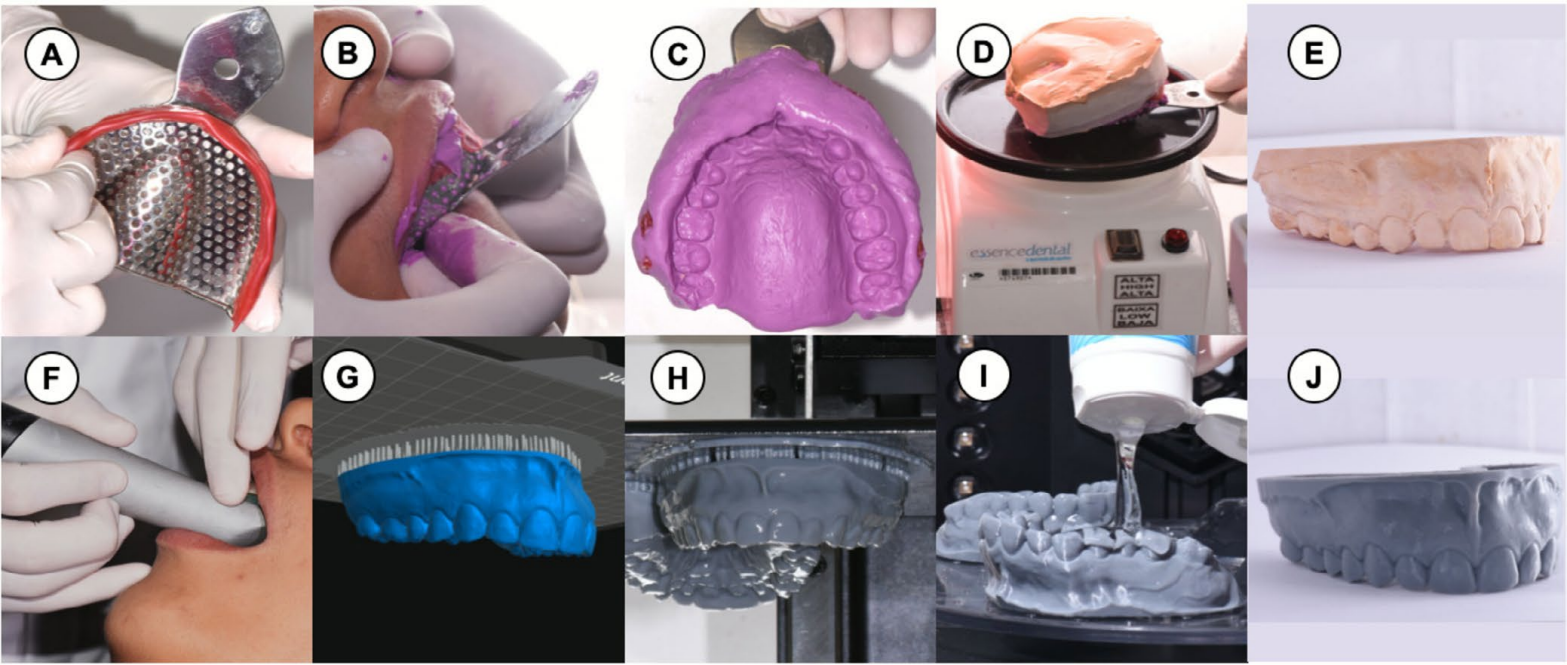
Molding protocols (conventional and digital): (A) Individualization of the mold; (B) Conventional mold taking; (C) Alginate impression obtained; (D) Pouring of the impression with type IV plaster; (E) Obtained dental stone cast; (F) Intraoral digital scanning; (G) Editing and designing of the digital model; (H) 3D‐printed resin model; (I) Application of water‐soluble gel to perform postcuring of the model; (J) 3D‐printed resin model obtained.

Conventional protocol—control (CMP): conventional impression using a metal impression tray (Bioart, Dental Equipment, São Carlos, SP, Brazil). The impression tray was individualized with utility wax on the edges to better delimit the impression areas (Figure 2A). Alginate (Hydrogum 5, Zhermack, Italy) was used and manipulated following the manufacturer's instructions. The powder was extracted from the package with a measuring spoon. The maxilla was molded, waiting 2 min for the material to set in the mouth (Figure 2B).

The impression was disinfected by washing with 4% chlorhexidine and, after a period of no more than 60 min, it was poured with type IV dental stone (Elite Rock, Zhermack, Italy), following the manufacturer's recommendation (Figure 2C). The dental stone was mixed in proportions using a vacuum system (Twister Evolution, Renfert, Hilzingen, Germany), adjusted to 350 rpm (mixing speed) and 95% vacuum for 30 s. The dental stone was poured under mechanical vibration (Vibramaxx, Essence Dental, Araraquara, SP, Brazil) (Figure 2D). The model was removed from the tray after 60 min and left to crystallize for 2 h in room temperature. The cut, shape, and design of the dental stone were standardized [13]. The model angles formed between the vestibular surface of the central incisor and the base of the working model were fixed at 90°, a height of 25 mm at the incisal edge of the upper central incisor, and a height of 20 mm at the mesiobuccal cusp of the upper first molar (Figure 2E).

Digital protocol (DMP): digital scanning of the maxilla was performed using an intraoral scanner (Straumann, Virtuo Vivo, Basel, Switzerland) copying all the teeth and the alveolar crest for the upper arcade, extending to the limits in the palate region beyond 5 mm from the gingival margin (Figure 2F). The stereolithography (STL) file was imported and processed in a workflow software (Exocad GmbH, Darmstadt, Hessen, Germany) generating the model. Then, the standardized STL file was imported into a 3D printing preprocessing software (ChiTuBox, V 2.1.0, Shenzhen, China).

The model was positioned in the center of the platform area and the printing supports were created (Figure 2G). The print settings were as follows: layer height 0.05 mm, base layer count 6, exposure time 2.6 s, and base exposure time 40 s. A UV‐sensitive 3D‐printed resin (photocurable at 405 nm) (Aqua‐Gray 4 K, Phrozen, Hsinchu City, Taiwan) was used in a 3D printer (Sonic Mighty 8 K, Phrozen) to manufacture the 3D‐printed resin model (Figure 2H). The model washing process was performed for 1 min in the washing station (Wash & Cure Kit) following the manufacturer's instructions, and subsequently dried for 30 min before postcuring in the Drying Station (Wash & Cure Kit, Phrozen). A water‐soluble clear gel coat (KY Jelly, Johnson & Johnson, New Jersey, USA) was applied to the entire surface of the model, with a thickness of 3 mm (Figure 2I), and the postcuring process was performed for 20 min using the ultraviolet light curing unit (Wash & Cure Kit, Phrozen) (Figure 2J) [12].

After each impression protocol, the athlete answered the comfort and satisfaction questionnaire based on the visual analogue scale (VAS). The questionnaire involved 7 questions according to their perception of duration, discomfort, anxiety level, nausea, pain, breathing difficulty, and unpleasant taste during the impression procedures. A scale from 0 to 10 cm was used, where 0 indicated “shorter duration or no discomfort” and 10 indicated “longer duration or a high discomfort” [39].

To assess the accuracy of the two working models obtained, conventional dental stone and 3D‐printed resin, for each patient, both models were evaluated using linear measurements of the width of the central incisors, canines, and first molars, as well as transverse dimensions including intercanine distance (ICD) and intermolar distance. These measurements were taken using a digital caliper (Mitutoyo, Tokyo, Japan), and the values obtained were compared between the two models [40].

**FIGURE 3:**
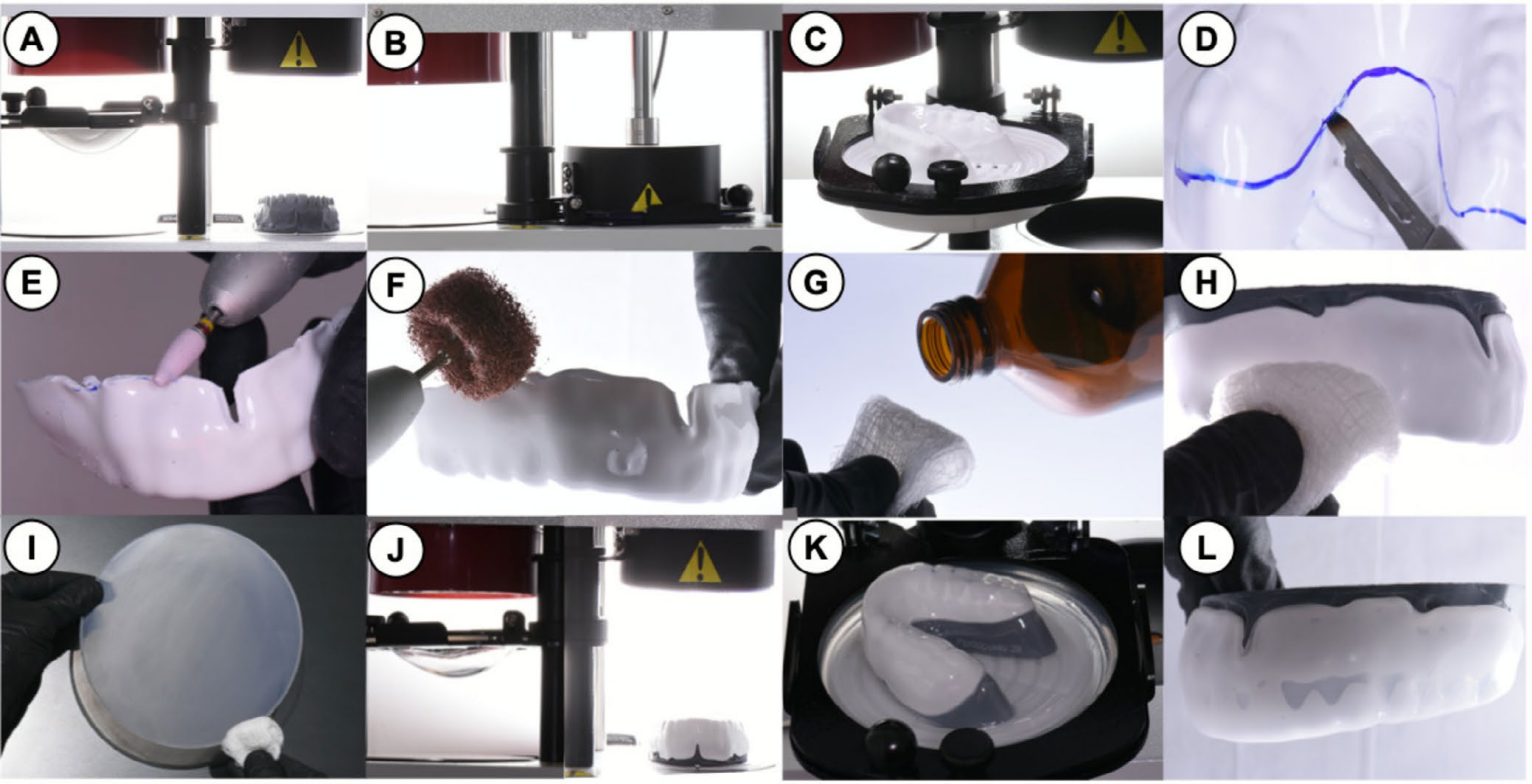
Mouthguard manufacturing protocol: (A) Heating of the first EVA plate for 314 s, at 137°C ± 5°C, using a positive pressure thermoforming machine; (B) Pressure/vacuum for thermoforming the EVA for 27 s; (C) Obtaining the first thermoformed plate and cooling for 45 min; (D) Scalpel cutting of the first plate of the mouthguard; (E) Finishing with a zirconia bur; (F) Polishing with American Burrs brushes; (G) Self‐curing acrylic resin monomer; (H) Active application of the monomer on the surface of the first thermoformed EVA plate; (I) Surface treatment of the 2nd EVA plate with monomer for 1 min; (J) Heating for 314 s of the 2nd EVA plate and plasticization on the 1st plate; (K) Obtaining thermoformed plates and cooling for 45 min; (L) Final appearance of the custom mouthguard.

Two 3 mm thick soft EVA plates (Bioart Dental Equipment, São Carlos, SP, Brazil) were used to obtain a 4 mm thick mouthguard. The first EVA plate was heated using a positive pressure rolling mill (Evolution Press, Essence Dental VH, Araraquara, SP, Brazil) for 314 s, at 137°C ± 5°C, until a uniform deformation of 2.5 cm (Figure 3A). The working model was centered on the pressure/vacuum platform. The pressure/vacuum was held for 27 s (Figure 3B). The mouthguard was cooled for 45 min at room temperature (22°C) before being removed from the model to avoid permanent deformations (Figure 3C).

**FIGURE 4:**
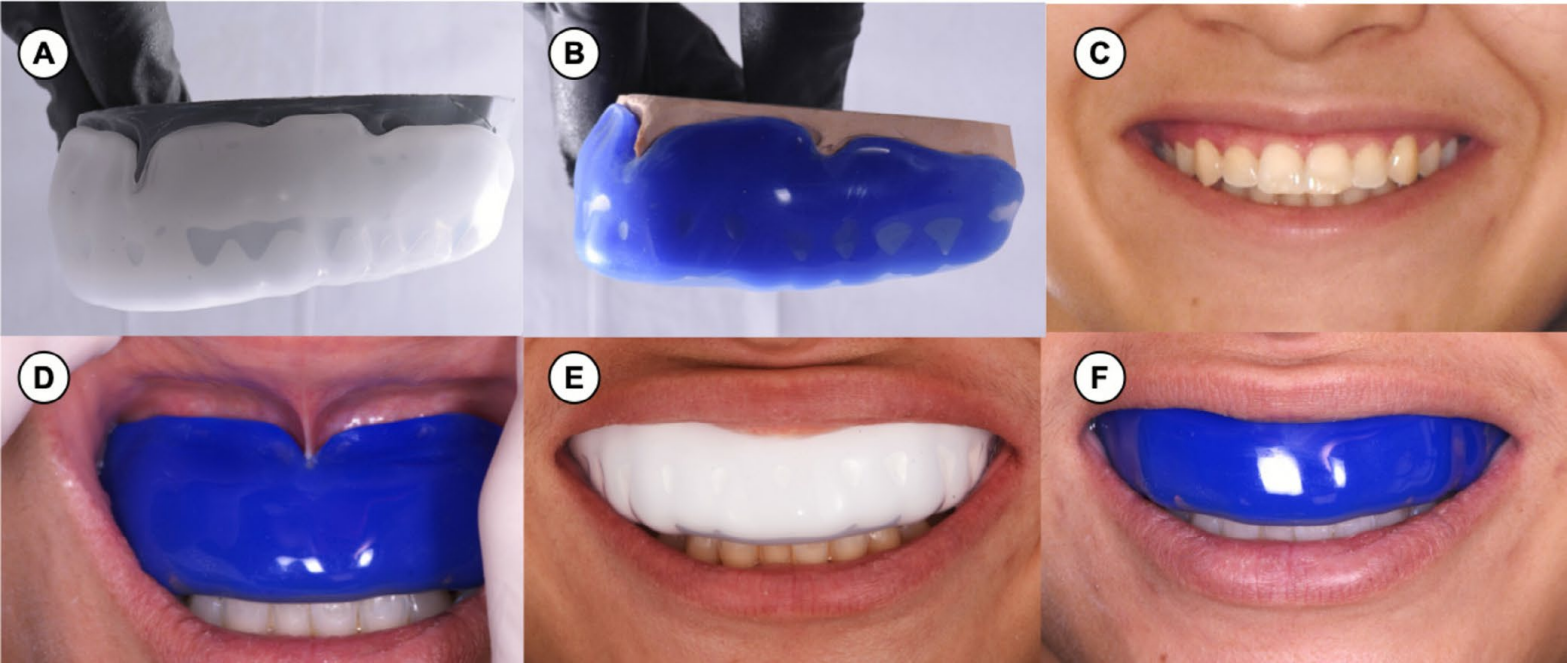
Initial adaptation and adjustment of the mouthguards obtained: (A) mouthguard obtained using the digital protocol—DMP‐mouthguard; (B) mouthguard obtained using the conventional protocol—CMP‐mouthguard; (C) Extraoral view of the patient's smile without the mouthguard; (D) Intraoral evaluation of mouthguard adaptation and occlusal adjustment; (E) Extraoral view of the smile with the adjusted DMP‐mouthguard; (F) Extraoral view of the smile with the adjusted CMP‐mouthguard.

The mouthguard border was cut using a heated scalpel blade no. 15 (Figure 3D), and finished using a zirconia burr (ZiBur, American Burrs, Palhoça, SC, Brazil) (Figure 3E) and polished using brushes (American Burrs) (Figure 3F). For the surface treatment with EVA, self‐curing acrylic resin monomer (Jet Clássico, São Paulo, Brazil) (Figure 3G) was applied actively to the surface of the first thermoformed EVA plate (Figure 3H) and on the inner surface of the second EVA plate for 1 min (Figure 3I) [14]. The second plate was heated for 314 s and molded onto the first layer of EVA in the model, and the same steps as for the previous layer were followed (Figure 3J,K). The mouthguard was stored over the model at room temperature (22°C) (Figure 3L). The athletes were randomly divided into two groups to decide which type of mouthguard each participant would receive first: the mouthguard made by the digital protocol (DMP‐MTG) (Figure 4A) or the one made by the conventional protocol (CMP‐ MTG) (Figure 4B). The participants and the operators who performed the adaptation and the assessment of the mouthguard were blinded to the protocol, type, and order of mouthguard use for each participant (double blinded protocol).

In the second session, the first mouthguard was adapted, adjusted, and installed (Figure 4C). The adaptation time and occlusal adjustment of the mouthguard were measured. For the initial adaptation of the mouthguard, the patient was asked to place it in the mouth, bite gently, and indicate if it was painful in any area. After indicating the area, the patient removed the mouthguard and handed it to the operator for adjustment and relief of the areas where there was discomfort (Figure 4D). For the occlusal adjustment, the mouthguard was heated and placed back in the mouth, biting down until all teeth evenly touched the guard (Figure 4E). Excess EVA around the bite marks was adjusted.

Intraoral and extraoral clinical photographs of the patient using the first mouthguard were taken to ensure a 5 min usage time before the immediate assessment (Figure 4F). Satisfaction and patient experience with the use of the first mouthguard were evaluated through a questionnaire based on the 10 cm VAS scale, where 0 indicated “none” discomfort or interference and 10 indicated “maximum discomfort or interference” [9, 27]. The questionnaire contained 12 assessment items: level of discomfort, misfit, instability, interference with speech, nausea, interference with breathing and performance, bullying, interference with swallowing, protective capacity, satisfaction, and esthetics while using the mouthguard.

**FIGURE 5:**
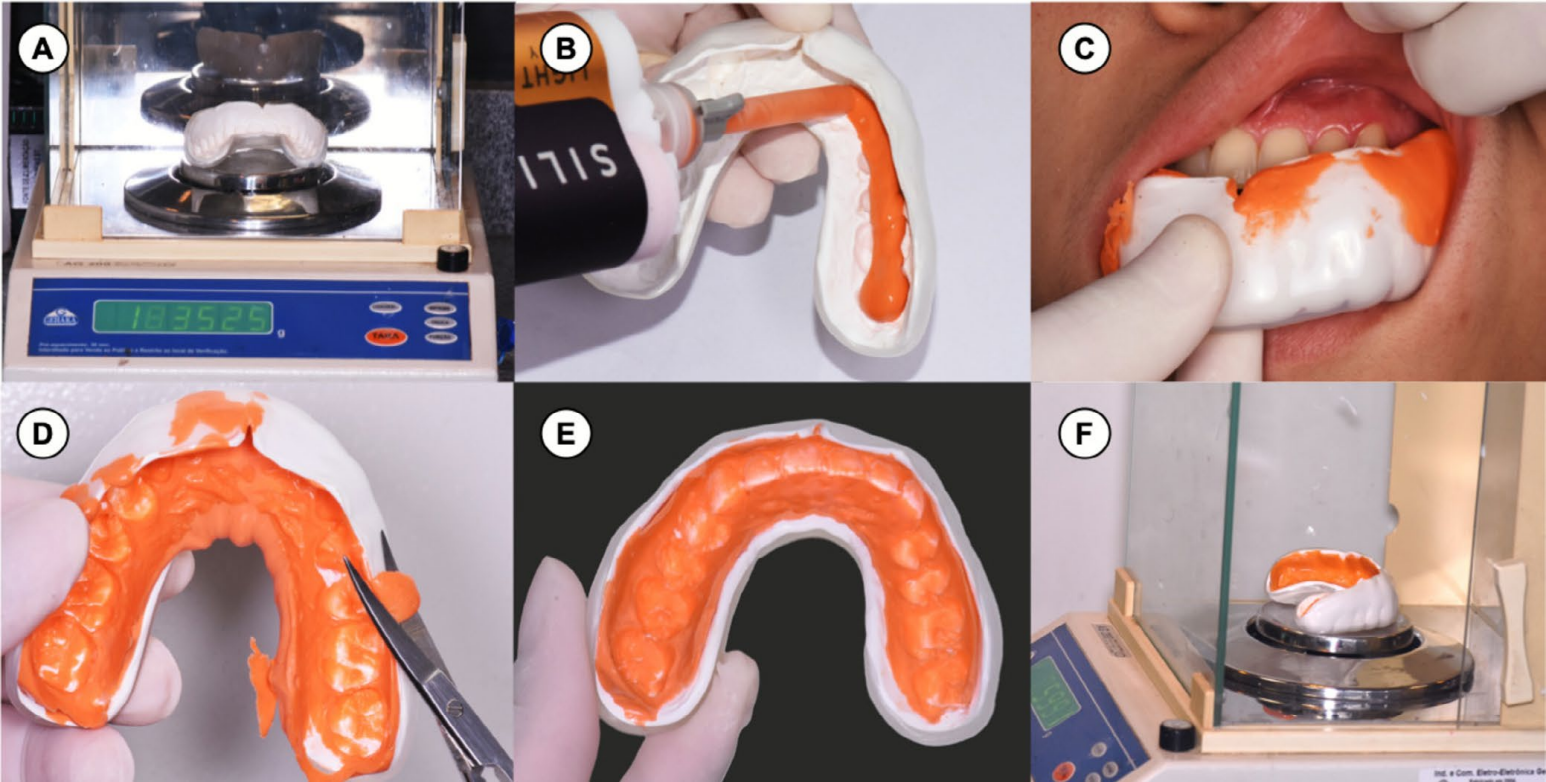
Assessment of mouthguards adaptation: (A) Initial weighing of the DMP‐mouthguard without silicone; (B) Application of addition silicone to the internal surface of the DMP‐mouthguard; (C) Placement of the silicone‐lined mouthguard in the maxilla for 3 min with light digital pressure; (D) Trimming of excess silicone from the DMP‐mouthguard; (E) Final appearance of the DMP‐mouthguard with silicone; (F) Final weighing of DMP‐mouthguard with silicone.

The adaptation was evaluated measuring the weight of the mouthguard (AG 200, Gehaka, São Paulo, SP, Brazil) before and after injection of the polyvinylsiloxane impression material (SilicOne Light Body, FGM, Joinville, SC, Brazil) between the inner surface of the mouthguard and the maxilla (Figure 5) [41].

The athletes received instructions about the use, care, hygiene, and storage of the first mouthguard during the 3 months. It was explained that they should use the mouthguard in all their training sessions and sports competitions. It was recommended that the mouthguard be washed with cold tap water and brushed gently with toothpaste and a toothbrush before and after each use. The participants were examined after 7 days, 1 and 3 months, to adjust the mouthguard if necessary and to fill in the same satisfaction and experience questionnaire.

After 3 months of mouthguard use, the participants returned to the Dentoalveolar Trauma Clinic for follow‐up evaluations and completed the 90‐day assessment questionnaire regarding the first mouthguard. Subsequently, the second mouthguard was adapted, adjusted, and delivered following the same procedures used for the first mouthguard (Figures 4 and 5). At the end of the appointment, athletes tested both mouthguards again and were asked: *“*Which mouthguard do you find more comfortable? And which one do you feel fits better in your mouth?” The fit of the mouthguard was recorded considering: 1. if the mouthguard stays securely in place when the athlete opens their mouth or moves their head, without having to clench their teeth. 2. If the mouthguard maintains stability when the athlete moves their tongue; 3. If the athlete experienced any discomfort, soreness, or pressure on any specific teeth or areas of their gums. To avoid any potential bias, the first evaluated mouthguard was retained at the clinic. The second mouthguard was then delivered, along with detailed instructions for use and assessment over a three‐month period, according to the previously described protocol.

To compare comfort and adaptation of the mouthguards, the McNemar test was used. Normality was verified by the Shapiro–Wilk test. The Wilcoxon test was applied for paired samples. The two‐way repeated measures ANOVA and the Holm‐Sidak method were used for multiple comparisons. Data analysis was performed using SigmaPlot v.12.0 software (Systat Software Inc., San Jose, USA), using a significance level of *α* = 0.05.

## 3 Results

The recruitment period took place between April and July 2024. Impression protocols were conducted from August to September 2024. The adaptation and initial adjustment of the first mouthguard occurred from October to November 2024, followed by a three‐month evaluation period. The second mouthguard was adapted and adjusted between January and February 2025, with its three‐month evaluation completed in May 2025.

Figure 1 presents the participant flowcharts according to the CONSORT guidelines, detailing the 76 athletes initially invited, the eligibility screening, and those who consented and attended consultations. The final sample consisted of 46 participants, as two athletes withdrew after the first stage and did not return. Table 1 summarizes the demographic characteristics, showing a predominance of males (80%) compared with females (20%). The age range was from 16 to 52 years, with the most common age group being 21 to 25 years (33%). Jiu‐Jitsu was the most frequently reported sport (61%). Facial or dentoalveolar trauma was reported by 41% of participants. The most commonly affected areas included the maxillary teeth (28%). The previous use of mouthguard was reported by 76% of participants. The thermoplastic “boil‐and‐bite” mouthguard was the most used type (57%). The overall mean number of training hours per week for all sports was 4.2 ± 1.8 h. The mean number of training sessions per week was 2.8 ± 0.9.

**TABLE 1:** General characteristics and history of athletes.

Variable	Category	Frequency	%
Gender	Male	37	80
	Female	9	20
Age (years)	16–20	4	9
	21–25	15	33
	26–30	10	22
	31–35	6	13
	36–39	3	7
	40–52	8	17
Active sport	Jiu‐jitsu	28	61
	Rugby	10	22
	Boxing	7	15
	Muay Thai	7	15
	Judo	7	15
	Kickboxing	5	11
	Wrestling	3	7
	Volleyball	2	4
	Basketball	1	2
	Handball	1	2
	Soccer	1	2
	MMA	1	2
Experience of dental trauma	Yes	19	41
	No	27	59
Injury/affected area	Maxillary teeth	13*	28
	Lips	8*	17
	Nose	5*	11
	Chin	6*	13
	Mandibular angle	4*	9
	Forehead	4*	9
	Eyes	2*	4
	Cheek	1*	2
	Lower teeth	2*	4
	Cheekbones	1*	2
	Eyebrows	2*	4
	No injuries/Could not identify	17*	37
Experience in using Mouthguard	Yes	35	76
	No	11	24
Mouthguards type used	Thermoplastic (boil and bite)	26*	57
	Stock	13*	28
	Custom‐fit	6*	13
	Not applicable	11*	24

* The inconsistency in comparing the total value of some items is due to the fact that participants could respond with multiple options, for example, in the affected area.

As presented in Table 2, digital impressions were associated with significantly lower levels of discomfort (*p* = 0.018), anxiety (*p* = 0.008), nausea (*p* = 0.027), breathing difficulty (*p* < 0.001), and unpleasant taste (*p* = 0.006). No significant difference was observed between protocols regarding pain perception (*p* = 0.971). Participants also perceived the digital protocol as shorter in duration compared to the conventional one (*p* = 0.023). Both methods produced clinically comparable models with respect to linear measurements (*p* > 0.05; Table 3). As shown in Table 4, the conventional impression protocol required significantly more time than the digital protocol (10:05 vs. 5:46 min; *p* < 0.001), consistent with participants' perception of procedure duration.

**TABLE 2:** Comparison of subjective parameters between digital and conventional impression protocols for producing mouthguards.

Parameter	Digital (Median [25%–75%])	Conventional (Median [25%–75%])	*p*
Perception duration time	0.5 [0.30–1.05]	0.8 [0.30–1.65]	0.023*
Discomfort	0.8 [0.40–1.30]	1.3 [0.55–2.85]	0.018*
Anxiety	0.4 [0.15–0.60]	0.5 [0.30–0.95]	0.008*
Nausea	0.4 [0.10–0.62]	0.5 [0.25–0.95]	0.027*
Pain	0.4 [0.20–0.80]	0.4 [0.25–0.75]	0.971
Difficulty breathing	0.5 [0.10–0.90]	1.0 [0.40–2.55]	< 0.001*
Unpleasant taste	0.4 [0.05–0.55]	0.5 [0.25–1.32]	0.006*

* Express significant difference (*p* < 0.05).

**TABLE 3:** Comparison of cross‐sectional dimensions (mm) obtained from conventional and digital models.

Measurement	Conventional	Digital	*p*
Width of central incisor 11	8.5 ± 0.5	8.5 ± 0.5	0.962
Width of central incisor 21	8.6 ± 0.6	8.6 ± 0.6	0.962
Width of canine 13	7.9 ± 0.4	7.9 ± 0.4	0.985
Width of canine 23	7.8 ± 0.4	7.8 ± 0.4	0.883
Width of first molar 16	9.9 ± 0.5	9.9 ± 0.5	0.997
Width of first molar 26	10.0 ± 0.5	10.0 ± 0.5	0.936
Intercanine distance	36.5 ± 2.2	36.4 ± 2.2	0.904
Intermolar distance	54.1 ± 2.8	54.1 ± 2.8	0.986

**TABLE 4:** Comparison of clinical times and difference in mouthguard (MTG) weights produced by conventional (CMP‐MTG) and digital (DMP‐MTG) protocols for adaptation assessment.

Parameter	Group/Technique	Mean ± SD	SEM	*p*
Molding protocol time	Conventional	10:05 ± 03:39	00:00:32	< 0.001
	Digital	05:46 ± 01:58	00:00:17	
Initial adaptation time in the mouth	CMP	07:12 ± 05:00	00:00:44	0.764
	DMP	07:12 ± 06:23	00:00:56	
Occlusal adjustment time	CMP	08:38 ± 04:49	00:00:43	0.509
	DMP	10:05 ± 08:11	00:01:12	
Mouthguard weight difference (g)	CMP	1.5 ± 0.5	0.08	< 0.001
	DMP	1.9 ± 0.7	0.11	

No significant differences were found between the mouthguard groups in terms of the clinical time required for initial adaptation or occlusal adjustment. However, a significant difference was observed in the intraoral adaptation of the mouthguards based on the weight variation between each mouthguard (*p* < 0.001), with the DMP‐mouthguard group showing greater weight variation compared to the CMP‐mouthguard group (Table 4). Perceived comfort did not differ significantly between the groups (*p* = 0.461; Table 5), although the CMP‐mouthguards were considered to be better fitting by the majority of participants (*p* = 0.027) compared to the DMP‐mouthguards.

**TABLE 5:** Comparison between mouthguards (MTGs) made by conventional (CMP) and digital (DMP) protocol in relation to comfort and fit (*n* = 46).

Evaluation Question	Types of MTGs	*n*	%	*p*
Which one is more comfortable?	CMP	20	43.9	0.461
	DMP	26	57.1	
Which one fit better?	CMP	31	68.1	0.027*
	DMP	15	31.8	

* Statistically significant (*p* < 0.05).

**FIGURE 6:**
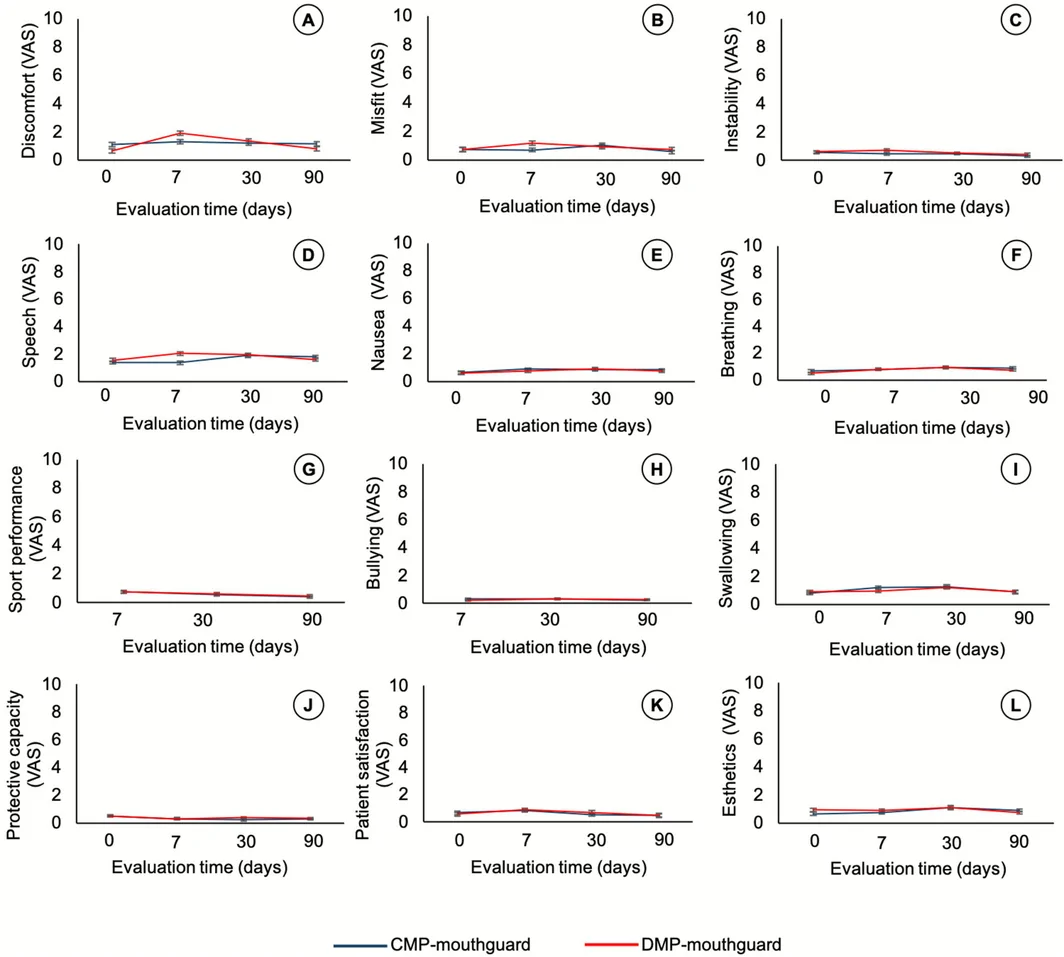
Comparison of means in the evaluation of athletes' experience of using mouthguards over time, based on 12 assessment items measured with the VAS scale: (A) Level of discomfort; (B) Perceived misfit; (C) Instability; (D) Speech interference; (E) Nausea; (F) Breathing interference; (G) Performance interference; (H) Bullying perception; (I) Swallowing interference; (J) Perceived interference with protective capacity; (K) Interference in satisfaction; (L) Esthetic interference during mouthguard use.

Adjusted means indicated low discomfort levels for both CMP‐mouthguard (1.2) and DMP‐mouthguard (1.2), with no significant difference between them. Discomfort peaked at 7 days (1.6), declining at 30 days (1.3), and reaching lower values at the immediate (0.88) and 90‐day (1.0) time points, suggesting a transient increase following the first week (Figure 6A).

Within‐group analysis showed stable discomfort over time in the CMP‐mouthguard group (*p* > 0.05), whereas the DMP‐mouthguard group exhibited a significant increase at 7 days compared to other time points (*p* < 0.05). Between‐group comparisons revealed that DMP‐mouthguard caused less discomfort immediately postadaptation (*p* = 0.034), but more discomfort at 7 days (*p* = 0.011); no significant differences were found at 30 or 90 days (Table 6). Despite these variations, both mouthguard types were well tolerated, with maximum discomfort levels remaining below 2 cm on a 10 cm scale, indicating acceptable comfort throughout the study period.

**TABLE 6:** Pairwise multiple comparisons of discomfort levels of CMP and DMP mouthguards (VAS scale) (Holm‐Sidak method, *α* = 0.05).

Comparison group	Mean difference	*t*‐value	*p* (unadjusted)	Critical level
**Inside the CMP**				
7 days vs. Immediate	0.21	0.90	0.376	0.009
7 days vs. 90 days	0.20	0.83	0.406	0.010
7 days vs. 30 days	0.12	0.50	0.618	0.013
30 days vs. Immediate	0.09	0.39	0.698	0.017
30 days vs. 90 days	0.08	0.33	0.740	0.025
90 days vs. Immediate	0.01	0.06	0.956	0.050
**Inside the DMP**				
7 days vs. Immediate	1.25	5.30	< 0.001	0.009*
7 days vs. 90 days	1.10	4.66	< 0.001	0.010*
30 days vs. Immediate	0.69	2.95	0.003	0.013*
7 days vs. 30 days	0.55	2.35	0.020	0.017
30 days vs. 90 days	0.54	2.31	0.022	0.025*
90 days vs. Immediate	0.15	0.64	0.524	0.050
**Between Mouthguards**				
CMP vs. DMP (Immediate)	0.47	2.14	0.034	0.050
DMP vs. CMP (7 days)	0.57	2.59	0.011	0.050
DMP vs. CMP (30 days)	0.13	0.60	0.546	0.050
CMP vs. DMP (90 days)	0.33	1.52	0.131	0.050

* Significant difference (*p* < 0.05).

No significant differences in perceived misfit were observed between CMP‐mouthguard and DMP‐mouthguard (*p* = 0.239). However, perceived misfit varied significantly across evaluation times (*p* = 0.031), peaking at 7 and 30 days and decreasing at the immediate and 90‐day assessments. This transient increase may reflect the need for fit adjustments during the early period of use, with improved adaptation noted by 90 days. No interaction was found between mouthguard type and time (*p* = 0.189), suggesting a similar adaptation (Figure 6B).

Regarding instability, no significant differences were detected between DMP‐mouthguard and CMP‐mouthguard (*p* = 0.145), nor across the different time points (*p* = 0.078), although a slight decrease in perceived instability was noted after 30 and 90 days (Figure 6C). The interaction effect was also not significant (*p* = 0.416), indicating consistent stability perception over time for both types of mouthguards.

Speech interference remained low for both mouthguards (≈2/10). A significant type‐by‐time interaction was detected (*p* = 0.006), driven by greater interference with the DMP‐mouthguard at 7 days (*p* = 0.002); no differences were found at the other time points (Figure 6D).

Nausea scores were uniformly minimal (≈0.9/10) with no significant effects of mouthguard type (*p* = 0.670), evaluation time (*p* = 0.488), or their interaction (*p* = 0.736) (Figure 6E). These findings indicate good tolerability of mouthguards with respect to that specific parameter.

Respiratory interference exhibited no type effect (*p* = 0.402) or interaction (*p* = 0.496) but varied over time (*p* = 0.050), peaking at 30 days relative to the immediate assessment (*p* = 0.006) and subsiding by 90 days, possibly reflecting neuromuscular adaptation. Absolute values remained low throughout, indicating minimal impact on breathing (Figure 6F).

Performance interference did not differ significantly between mouthguard types (*p* = 0.577), with similar mean scores for both mouthguards, progressively decreasing until 90 days, more notably in the digital group. A significant temporal effect was observed (*p* = 0.018), with greater interference at 7 days compared to 90 days (*p* = 0.005), suggesting a temporary impact probably related to neuromuscular adaptation. No significant interaction was found between mouthguard type and time (*p* = 0.899) (Figure 6G).

Perceived bullying did not differ significantly between mouthguard types (*p* = 0.709), time points (*p* = 0.557), or their interaction (*p* = 0.417). Mean scores remained low and stable over time for both CMP‐mouthguard (0.31) and DMP‐mouthguard (0.28), indicating minimal social discomfort (Figure 6H).

Swallowing interference did not differ significantly between mouthguard types (*p* = 0.614), nor was there a significant interaction between type and evaluation time (*p* = 0.458). However, a significant effect of time was observed (*p* = 0.037), with a slight increase at 30 days (Figure 6I). Although this temporal variation was not confirmed by pairwise comparisons, it may be associated with breathing interference due to the neuromuscular adjustment process. Low mean values indicated minimal interference and comparable behavior of both models over time.

No significant differences were found between CMP‐mouthguard and DMP‐mouthguard regarding interference with protective capacity (*p* = 0.224), nor in the interaction between mouthguard types and time (*p* = 0.507). A significant effect of time was observed (*p* = 0.003), with greater interference reported immediately after fitting compared to later evaluations (*p* < 0.01). Mean interference values remained low throughout (Figure 6J), indicating mild functional impact that decreased as users adapted to the mouthguards.

Similarly, interference with satisfaction showed no significant difference between mouthguard types (*p* = 0.829) but varied significantly over time (*p* = 0.043), reflecting neuromuscular adaptation. No significant interaction between type and time was found (*p* = 0.686). Peak interference occurred at 7 days (mean = 0.87), decreasing by 90 days (mean = 0.487), with a significant difference between these points (*p* = 0.005) (Figure 6K). Overall low scores near zero on a 0–10 scale indicate favorable acceptance of both mouthguards.

Regarding interference with esthetics, no significant differences were observed between mouthguard types (*p* = 0.657), across evaluation times (*p* = 0.062), or in the interaction between type and time (*p* = 0.355). Mean values were similar for both models, CMP‐mouthguard = 0.86; DMP‐mouthguard = 0.92, and remained stable throughout the evaluation period (Figure 6L). These low scores on a 0–10 cm scale indicate favorable esthetic acceptance of both mouthguards.

## 4 Discussion

A significant difference was identified between the two types of mouthguards, especially with regard to mouthguard adaptation, subjective comfort, and the evaluation of usability through functional parameters; therefore, the null hypothesis was rejected. For the other parameters, no statistically significant difference was observed.

Impression time differed significantly between methods, with the digital workflow protocol being notably faster in producing mouthguards. Reducing appointment time is an advantage of digital dentistry and can increase clinical efficiency and patient acceptance, especially in sports practice, when efficiency is crucial [42]. The digital workflow eliminates discomforts commonly associated with conventional impressions, such as choking and respiratory difficulties [43]. This aspect was reported by the athletes, who indicated less discomfort with the use of mouthguards produced by the digital workflow. These aspects specifically involve the perception of procedure duration, discomfort, anxiety, nausea, respiratory difficulties, and unpleasant impression material taste. However, the pain reported was low and similar for both mouthguards, demonstrating no clinical relevance concerning mouthguard production. The accuracy of the working models was similar for both methods, indicating that mouthguard adaptation was not influenced by changes in model dimensions.

Both types of mouthguards showed satisfactory dimensional stability and adequacy in terms of coverage of dental structures, compatible with the recommendations for orofacial protection. Previous studies have highlighted that a better fit leads to greater comfort and increased adherence to the mouthguard use [11, 13, 44]. Although both mouthguards tested in this study showed adequate adaptation and adjustment to prevent displacement during sports practice, the DMP‐mouthguard presented a greater internal adaptation gap. The differences can be attributed mainly to the material used to obtain each working model, since previous studies have demonstrated the good conductivity of dental stone for heat transmission, in contrast to the resin material of the printed models [12, 45, 46, 47], which, being a polymeric material, has low thermal conductivity, and this may have interfered with the detailed copying of the model surface during plasticization and EVA aspiration. This difference in adaptation influenced the athletes' comfort; they reported that the DMP‐mouthguard was more comfortable because it was less tight than the CMP‐mouthguard. This was more frequently reported as a satisfactory feature by athletes who trained for longer periods.

Another important aspect revealed by this study is the temporal adaptation process experienced by athletes using both mouthguard types. While initial comfort was slightly higher for DMP‐mouthguards, a transient increase in discomfort was noted at 7 days, particularly for this group. This may reflect a period of muscular and occlusal adaptation, as users acclimate to the feel and function of the appliance during physical exertion. The subsequent reduction in discomfort at 30 and 90 days supports the notion that this effect is temporary and self‐limiting. Such findings are consistent with existing literature suggesting that even well‐fitted intraoral mouthguards may initially provoke discomfort due to increased oral awareness and neuromuscular adaptation [48, 49]. Clinically, this highlights the importance of patient education and follow‐up in the early phase of mouthguard use, particularly for athletes new to custom‐fit mouthguards.

One limitation of this study was the restricted diversity of sports represented. Most participants were engaged in contact sports, particularly Brazilian Jiu‐Jitsu and Rugby, both of which carry a high risk of orofacial trauma due to direct physical contact and impacts. The limited participation of athletes from other high‐risk sports prevented meaningful comparisons across different discipline‐specific demands regarding the use of custom‐fit mouthguards. It is important to note that athletes in noncontact or semicontact sports, such as sprinters, soccer players, or other speed‐based disciplines, are also vulnerable to dental injuries caused by falls, collisions, or accidental impacts during intense physical activity. These groups may present distinct functional requirements, including unobstructed breathing and minimal speech interference, which can directly influence mouthguard design and perceived acceptability [3, 9, 23]. This highlights the importance and relevance of evaluating custom‐fit mouthguards in a wider range of high‐risk sports, ensuring more representative and balanced participation across disciplines. Therefore, future research should include athletes from a broader spectrum of sports to guarantee that the protective, functional, and ergonomic features of mouthguards meet the diverse needs of all athletic profiles.

A clinical study conducted to evaluate the comfort and usability properties of custom‐fit mouthguards and boil‐and‐bite mouthguards in basketball players also demonstrated that custom‐fit mouthguards presented less interference with breathing, speech, and greater overall comfort than stock mouthguards [9]. In the present study, the method used influenced the athletes' performance; DMP‐mouthguards resulted in less interference in breathing, swallowing, and verbal communication during sports practice. Despite the differences in favor of DMP‐mouthguard, both models showed acceptable performance in functional dimensions. Based on the results obtained, the use of patient‐reported outcome measures is essential in clinical dental research. These tools enable precise assessment of patient experiences regarding discomfort, adaptation, and satisfaction with different impression and mouthguard fabrication techniques. Incorporating patient perception alongside objective clinical parameters provides a comprehensive understanding of the functional and psychological impacts of dental materials and procedures. This approach is critical for optimizing mouthguard design and treatment protocols, ultimately improving patient compliance and clinical outcomes. Therefore, integrating subjective patient feedback with technical evaluations is indispensable for advancing evidence‐based dental practice.

This study has some limitations regarding the objective clinical parameters used. No master model was employed to compare the accuracy of dental stone casts with 3D‐printed resin models, as this step was considered unnecessary; the stone model obtained through the conventional technique served as both the control and reference. Furthermore, the adaptation of the mouthguards was assessed solely by weight. Although this method was effective in detecting the presence or absence of internal misfit and quantifying the degree of misfit in grams, it did not allow precise millimetric measurements. Consequently, it was not possible to establish a minimum acceptable threshold of misfit in custom‐made mouthguards or to directly correlate this parameter with the athletes' subjective perceptions.

Future research should develop methods to precisely quantify the acceptable range of internal misfit in mouthguards without compromising their protective, functional, or comfort properties. Additionally, the potential of fully digital workflows, including direct 3D printing of the final devices, should be explored, as this could eliminate EVA thermoforming and reduce adaptation time and occlusal adjustments, minimizing negative effects on fit and comfort.

## 5 Conclusion

The digital workflow offered clinical advantages in terms of scanning time, perceived comfort, and functional performance during sports practice, without compromising the protective efficacy, accuracy, stability, or overall performance of the custom‐made mouthguard. The DMP‐mouthguard demonstrated favorable adaptation for athletes engaged in long‐duration training, as it minimized soft tissue compression, resulting in greater comfort without negatively impacting protective capacity. No relevant interference with sports performance was reported for either type of mouthguard, thereby reinforcing their clinical acceptance and functional integration into athletes' routines. These findings support the use of the digital workflow as a promising impression protocol for the fabrication of custom‐made mouthguards.

## Author Contributions

Maryuri Macías Bautista: experimental development, bibliographic research, data analysis, manuscript preparation and editing. Priscilla Barbosa Ferreira Soares: conception and design, data analysis, manuscript editing. Airin Karelys Avendaño Rondon: experimental development, bibliographic research, data analysis, manuscript preparation and editing. Laryssa Silva da Cunha: experimental development, bibliographic search, preparation and editing of manuscripts. Ianca Daniele Oliveira de Jesus: experimental development, bibliographic research, preparation and editing of manuscripts. Rodrigo Silva Moreira: experimental development, bibliographic research, preparation and editing of manuscripts. Gisele Rodrigues da Silva: conception and design, data analysis, manuscript editing. Liran Levin: conception and design, data analysis, manuscript editing. Carlos José Soares: conception and design, literature search, data analysis, obtaining funding, manuscript editing and manuscript review. All authors read and approved the final manuscript.

## Funding

This work was supported by Conselho Nacional de Desenvolvimento Científico e Tecnológico, INCT‐Saúde Oral e Odontologia # 406840/2022‐9. Fundação de Amparo à Pesquisa do Estado de Minas Gerais, APQ‐04262, APQ‐03238‐24, RED‐00204‐23. Coordenação de Aperfeiçoamento de Pessoal de Nível Superior, Finance code 001.

## Ethics Statement

This clinical trial adhered to the ethical principles of the Declaration of Helsinki and was approved by the local ethics committee (protocol number CAAE: 77752824.4.0000.5152) and registered in the Brazilian Clinical Trials Registry (UTN: U1111‐1316‐7546; URL: REBEC; date of registration: 17/03/2025).

## Conflicts of Interest

The authors declare no conflicts of interest.

## Supporting information


**Appendix S1:** edt70059‐sup‐0001‐AppendixS1.docx.

## Data Availability

The data that support the findings of this study are available on request from the corresponding author. The data are not publicly available due to privacy or ethical restrictions.
